# Molecular Diagnosis Is Vital to the Accurate Classification and Management of Thrombotic Thrombocytopenic Purpura in Children

**DOI:** 10.3389/fimmu.2022.836960

**Published:** 2022-04-11

**Authors:** Cecile L. Karsenty, Susan E. Kirk, Hannah L. Helber, Jose M. Esquilin, Jenny M. Despotovic, Amanda B. Grimes

**Affiliations:** ^1^ Department of Pediatrics, Section of Hematology/Oncology, Baylor College of Medicine, Houston, TX, United States; ^2^ Texas Children’s Cancer and Hematology Centers, Texas Children's Hospital, Houston, TX, United States; ^3^ Methodist Children’s Hospital, San Antonio, TX, United States; ^4^ Methodist Physicians Pediatric Specialists of Texas, San Antonio, TX, United States

**Keywords:** congenital TTP, immune-mediated TTP, pediatric, ADAMTS13, inhibitor

## Abstract

Thrombotic thrombocytopenic purpura (TTP) is a rare but potentially life-threatening hematologic disease, presenting a myriad of diagnostic and management challenges in children. Here, we provide a review of this disorder and discuss 2 exemplary cases of TTP occurring in adolescents, emphasizing the need for consideration of late-onset congenital TTP (cTTP). We demonstrate the importance of early confirmation of ADAMTS13 enzyme deficiency and the presence or absence of ADAMTS13 inhibitor in order to rapidly initiate the appropriate life-saving therapies. Ultimately, molecular testing is paramount to distinguishing between congenital and acquired immune-mediated TTP.

## Introduction

Thrombotic thrombocytopenic purpura (TTP) is a life-threatening hematologic disease, requiring prompt recognition and intervention. It is a rare cause of thrombotic microangiopathy characterized by microangiopathic hemolytic anemia (MAHA), severe thrombocytopenia and high risk for ischemic end organ damage secondary to formation of platelet-rich thrombi in the microvasculature (1, 2). These microthrombi occur in the setting of inadequately cleaved von Willebrand factor (VWF), caused by the absence or severe deficiency of ADAMTS13 (a disintegrin and metalloproteinase with a thrombospondin type 1 motif, member 13) activity. ADAMTS13 is a plasma protein first characterized in 2001, that under normal circumstances cleaves von Willebrand factor (VWF) into smaller multimers (3, 4). Prevention or reversal of end-organ ischemia in TTP is achieved by re-establishing adequate ADAMTS13 enzyme activity, and thereby appropriate VWF cleavage. TTP can be caused by an inherited deficiency of ADAMTST13 activity, resulting in congenital TTP (cTTP). More commonly, however, TTP is due to an acquired deficiency of ADAMTS13 activity resulting from autoantibody-mediated inhibition of plasma ADAMTS13 activity, referred to as immune-mediated TTP (iTTP). Classical forms of TTP may be more readily identified; but atypical presentations of either cTTP or iTTP present significant diagnostic and management challenges. In this review, we discuss the clinical presentation, diagnostic work up and most up to date therapeutic interventions for TTP, a rare but potentially fatal hematologic disease. We highlight the role of genetic testing to differentiate cTTP and iTTP. To illustrate potential diagnostic and therapeutic challenges, we will refer to two cases, both adolescents, presenting with acute hemolytic anemia and thrombocytopenia, ultimately diagnosed with TTP but requiring very different management strategies.

## Background

### Pathophysiology

**Figure 1:**
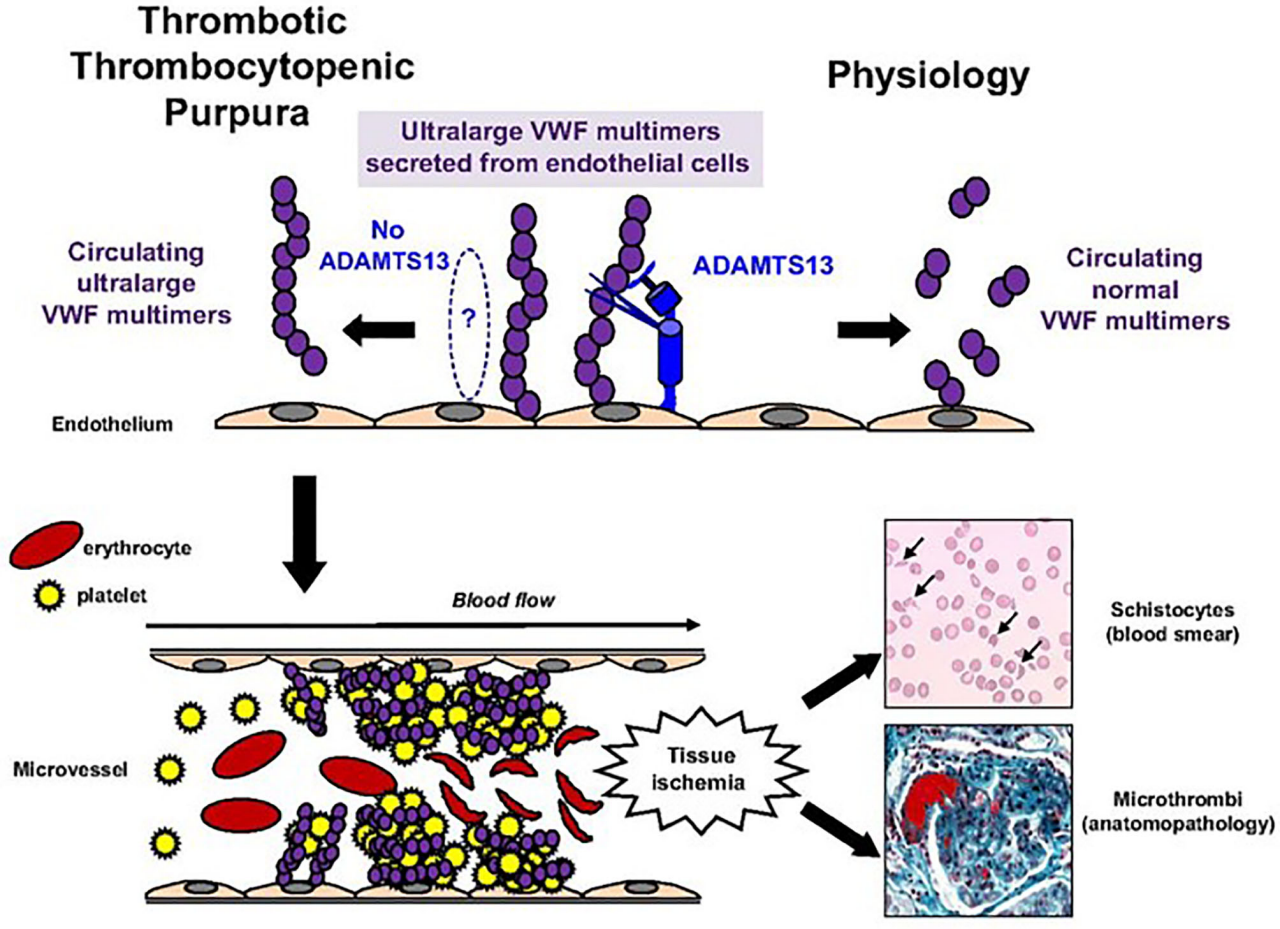
Pathophysiology of TTP. Pathophysiology of TTP. In physiologic conditions, ultralarge VWF multimers released from endothelial cells are cleaved by ADAMTS13 in smaller VWF multimers, less adhesive to platelets. In TTP, because of the absence of functional ADAMTS13 (either absent by congenital defect or inhibited by specific autoantibodies), ultralarge VWF multimers are released into the blood and bind spontaneously to platelets to form aggregates within the arterial and capillary microvessels. The VWF platelet aggregates are large enough to form microthrombi inducing tissue ischemia, platelet consumption, and microangiopathic hemolytic anemia (schistocytes on blood smear).This figure was originally published in *Blood*, and re-printed with permission here. Bérangère S. Joly, Paul Coppo, and Agnès Veyradier. Thrombotic thrombocytopenic purpura. *Blood.* 2017;129(21):2836-2846. ^©^ the American Society of Hematology.

TTP is a rare cause of thrombotic microangiopathy defined by clinical criteria, biological markers of intravascular hemolysis, and severe ADAMTS13 deficiency. Severe ADAMTS13 deficiency leads to accumulation and aggregation of platelets *via* ultra-large VWF multimers. Von Willebrand factor, a glycoprotein required for platelet adhesion, is secreted as ultra-large multimers and stored as such until released into circulation upon vascular injury or endothelial cell activation. Under normal physiological circumstances, ADAMTS13 in peripheral circulation facilitates proteolysis of ultra large VWF multimers into smaller forms to prevent abnormal or disorganized activation, with excessive platelet binding. In TTP, however, ADAMTS13 activity is absent or significantly decreased, leading to abnormal platelet adhesion and aggregation due to uncleaved, ultra large VWF multimers, causing formation of diffuse microthrombi within peripheral vasculature (5) (see **Figure 1**). In most cases (>95%), absence of ADAMTS13 activity is due to acquired autoantibodies against ADAMTS13, inhibiting its proteolytic activity. In a minority of patients (<5%), ADAMTS13 deficiency is due to biallelic mutations within the ADAMTS13 gene leading to an inherited chronic deficiency of ADAMTS13, termed congenital TTP (cTTP).

### Epidemiology

Global incidence of TTP is reported as 2 to 6 per million individuals with an overall female predominance. Immune mediated TTP (iTTP) represents the vast majority of TTP cases (>95%), generally presenting in late adolescence and early adulthood. Women are more frequently affected than men (~2 to 1). Possible risk factors or triggers that have been linked to iTTP include infections, medications, pregnancy, known autoimmune disease, or evidence of underlying autoimmunity (5). Congenital TTP (cTTP), also referred to as Upshaw-Schulman syndrome (USS), is a very rare autosomal recessive disorder caused by biallelic homozygous or compound heterozygous variants within the ADAMTS13 gene, leading to severely decreased or absent proteolytic activity. More than 200 ADAMTS13 variants across all ADAMTS13 protein domains on chromosome 9 have been identified in patients with cTTP (including missense, non-sense, splice site, and frameshift mutations). Clinical phenotype of cTTP can be quite heterogeneous. Classically, cTTP is thought to present early in childhood; however, more recent case studies have reported patients with milder or even absent clinical signs of disease in childhood but later developing clinically evident hemolysis or small vessel ischemia. Certain pathologic mutations, specifically p.R1060W homozygosity, have been shown to result in residual ADAMTS13 activity, around 5-10% of normal plasma levels, more frequently resulting in this ‘late onset’ cTTP (6). Like iTTP, active thrombotic microangiopathic events in these patients seem to be triggered by an additional exogenous or endogenous event such as medication use or a state of physiological stress, including pregnancy or infection (7). Importantly however, unlike those with iTTP, all patients with cTTP are at significantly increased risk for transient ischemic attack (TIA) and overt stroke starting at a young age, with up to 20% lifetime incidence of stroke given potential long term ADAMTS13 deficiency (8, 9).

Childhood-onset TTP, defined as initial MAHA episode before age 18, whether cTTP or iTTP, is a rare entity representing only about 5-10% of all TTP cases. A child presenting with an acute episode of TTP presents its own diagnostic and management challenges. However, it is important to note that a higher proportion of pediatric cases represent an initial episode of cTTP highlighting importance for molecular diagnosis. Based on data from the national Registry of the French Reference Center for thrombotic microangiopathies, approximately 35% of newly diagnosed TTP cases in childhood are attributed to cTTP and may present at any age, from neonate to adolescent (10). Pediatric acquired or immune mediated forms of TTP generally present in late teenage years (11). Mortality for untreated TTP, both in pediatric and adult patients, approaches 90%. However, if appropriate therapeutic interventions are initiated promptly at the time of diagnosis, overall mortality is <10%.

## Discussion

### Clinical Diagnosis

The clinical spectrum of TTP can vary widely; however, the vast majority if not all patients will present with severe thrombocytopenia (platelet count <30 x10^9/L), evidence of microangiopathic hemolytic anemia (including schistocytes on peripheral smear, elevated LDH, indirect hyperbilirubinemia, low haptoglobin, and elevated plasma free hemoglobin) along with potential evidence of end organ damage. Some estimates suggest that up to 60% of patients will have neurological symptoms at presentation, ranging from headache to overt stroke or seizures, and up to 25% may have evidence of cardiac or mesenteric ischemia. Renal manifestations however are typically isolated to proteinuria and/or hematuria, with acute renal failure an unusual finding at the time of diagnosis. Based on reports from the Oklahoma TTP registry, the majority of patients with acute iTTP present with a platelet count of <20 x10^9/L, hematocrit <30% and normal to minimally elevated serum creatinine, a finding particularly helpful when differentiating TTP from other forms of TMA, most notably hemolytic uremic syndrome (HUS) (12, 13). Because the signs, symptoms, and laboratory markers overlap with other thrombotic microangiopathies, the distinction of a TTP diagnosis relies on determination of ADAMTS13 activity (2, 14). This is the only biologic marker specific to TTP, and diagnosis is confirmed when ADAMTS13 activity is found to be <10%. However, ADAMTS13 activity levels are often not readily available at the time of presentation in a patient with evidence of MAHA; and in the vast majority of clinical scenarios, the clinician must decide to initiate risky but life-saving therapy before having laboratory confirmation of a TTP diagnosis.

Given this, several clinical scoring systems have been developed to overcome diagnostic challenges and treatment delays related to ADAMTS13 testing turnaround. The goal of these scoring systems – the Bentley score, the French score, and the PLASMIC score – is to identify severe ADAMTS13 deficiency based on the presence of significant clinical predictors alone (prior to confirmation of ADAMTS13 activity) (12, 15–18) (see **Table 1**). Although clinical scoring criteria were developed based on adult cohorts, these criteria may be applicable to pediatric patients presenting with TTP, particularly adolescent cohorts.

**Table 1:** Clinical Scoring Systems for the Prediction of Severe ADAMTS13 Deficiency (or TTP).

	Bentley score	French score	PLASMIC score
**Predictive Clinical Factors**	• indirect hyperbilirubinemia • elevated reticulocyte percentage • thrombocytopenia • normal or minimally elevated creatinine	• creatinine <2.26 mg/dL • platelet count<30 x10^9/L • positive ANA	• platelet count <30x10^9/L • no evidence of malignancy • MCV <90 fL • INR <1.5 • creatinine <2.0 mg/dL • evidence of hemolysis (reticulocyte count >2.5%, undetectable haptoglobin, indirect bilirubin >2 mg/dL)

We illustrate this diagnostic challenge, in absence of rapid ADAMTS13 testing, given widely variable and often evolving clinical presentation with two adolescent patient cases. The first patient, case 1, is that of a 17-year-old healthy male, with a family history notable for autoimmune disease, who initially presented to an outside hospital following a brief SARS-CoV-2 infection with hematuria, mild normocytic anemia (hemoglobin 11 g/dL), severe thrombocytopenia (platelet count <20 x 10^9/L), and easy bruising. He was diagnosed with presumed immune thrombocytopenic purpura (ITP) and treated with intravenous immunoglobulin (IVIG) x2, with minimal improvement in bleeding symptoms and platelet count. ADAMTS13 testing was sent but results were not available at time of discharge, and outpatient hematology follow up was planned.

The second patient, case 2, is a 16-year-old female with past medical history notable for polycystic ovarian syndrome, depression and obesity who initially presented to an outside emergency room with persistent gross hematuria and scattered petechiae. She was found to be severely thrombocytopenic with a platelet count <20 x10^9/L, and a mild normocytic anemia with hemoglobin 9.6 g/dL. She received IVIG therapy for presumed ITP. However, thrombocytopenia persisted, and anemia worsened, with hemoglobin decreasing to 7.6 g/dL over the next 48 hours, along with an increasing reticulocyte count, rising lactate dehydrogenase (LDH), and persistent hemoglobinuria on urinalysis. A bone marrow aspirate and biopsy showed no evidence of malignancy; and with increasing evidence of hemolysis and neurological abnormalities, a presumptive diagnosis of TTP was made. She was then transferred to a large pediatric tertiary care center, with capacity for in-house ADAMTS13 testing, where confirmatory testing was sent.

### ADAMTS13 Activity and Inhibitor Testing

ADAMTS13 activity of <10% is necessary to confirm the diagnosis of TTP and is also important in monitoring clinical response to therapy. Accurate measurement of both ADAMTS13 antigen levels (via ELISA) and functional ADAMTS13 activity [via fluorescence resonance energy transfer based assay (FRETS-VWF73) and collagen-binding activity assay] are crucial to the management of TTP. However, just as important to TTP management, is the identification of ADAMTS13 inhibitor. As demonstrated in the cases presented here, this testing is crucial to the distinction between iTTP and cTTP, and ultimately to providing the appropriate management for these patients.

In the case of iTTP, ADAMTS13 auto-antibodies may be either inhibitory or non-inhibitory antibodies, based on their ability to block proteolytic cleavage of VWF *in vitro*. Most patients will have inhibitory antibodies identified, generally IgG; however, 10-15% of iTTP patients have non-inhibitory antibodies that these assays will not detect (5). Traditional assays such as ELISA and FRETS-VWF73 are done *via* incubation of test samples with standard human plasma at varying concentrations, using a standard mixing study. These allow for identification of antibodies leading to ADAMTS13 inhibition *in vitro*. However, two major limitations of these panels exist: false negatives as well as false positives are possible, particularly with collagen-binding based inhibitor panels as they do not allow for identification of non-inhibitory or non-neutralizing antibodies. These are antibodies that do not lead to proteolytic inhibition *in vitro*; however, *in vivo* they lead to severe ADAMTS13 activity deficiency by affecting normal interaction with endothelium or other cellular and plasmatic modulators essential for *in vivo* activity (19–22). These nuances of inhibitor testing must be considered and results interpreted cautiously, with careful correlation to patient’s clinical phenotype.

The progression of case 1 illustrates this diagnostic complexity. After his initial presentation, he re-presented to the emergency room one week later with neurological symptoms including memory loss, lethargy, and cognitive slowing. Previously sent ADAMTS13 assay resulted at this time demonstrating undetectable activity (<10%). Inhibitor testing suggested the presence of protease inhibitor (0.9 Bethesda equivalent units, reference range <0.4). Given a high suspicion for iTTP he was immediately initiated on frontline therapy for iTTP (described below). However, he did not exhibit desired response, as he had no significant improvement in hemolytic anemia, thrombocytopenia, or overall functional status. Repeat inhibitor testing as his clinical course progressed (initially utilizing a mixing study, and later enzyme-linked immunoassay [ELISA]) ultimately showed no evidence of ADAMTS13 inhibitor presence. As a result, ADAMTS13 gene sequencing was done demonstrating presence of a homozygous, pathogenic variant *ADAMTS13* c.1584+5G>A (p.)?, which has been previously reported in association with clinical TTP. Parental testing confirmed the cTTP diagnosis, showing each parent to be a clinically unaffected heterozygous carrier of this ADAMTS13 gene variant. This single splice variant, previously reported by Levy et al. in 2001, results in markedly reduced or absent utilization of the normal intron 13 splice donor and activates a cryptic donor splice site at +70, resulting in a 23-codon insertion (3).

Conversely, ADAMTS13 testing for our second patient was notable for an enzyme activity level of 1%, confirming the clinical diagnosis of TTP. She also had a very high inhibitor identified at presentation, based on ELISA testing (88%, reference range <15%), suggestive of proteolytic inhibition likely from IgG autoantibody.

With more than 200 mutations associated with cTTP and clinically relevant limitations of *in-vitro* inhibitor testing as exhibited by case 1, molecular testing should be considered early on, particularly when patients are exhibiting atypical or poor response to immunosuppressive therapy. This is important to ensure rapid and appropriate therapeutic interventions, as treatment strategies for cTPP and iTTP are distinct.

### Treatment

Prompt initiation of medical therapy is crucial in preventing severe morbidity and mortality in TTP. First-line therapy for iTTP is therapeutic plasma exchange: clearing autoantibodies and providing ADAMTS13 in circulation; while ADAMTS13 replacement alone (with FFP, for example) may be utilized for cTTP upfront.

Prior to the advent and use of therapeutic plasma exchange (TPE), mortality rate from acute TTP was near 90%. Its initiation should not be delayed, as immediate TPE has been shown to decrease the risk of mortality related to acute TTP events to <10% (23). Experience and research have shown that in addition to TPE, incorporating adjunct immunosuppressive therapies leads to higher rates of remission and lower rates of relapse; most notably the use of glucocorticoids and rituximab for patients with iTTP (24–28). Upfront rituximab therapy in combination with corticosteroids and TPE therapy has been largely adopted as the front-line standard of care for iTTP (29).

For patients with iTTP, TPE allows for removal of ADAMTS13 autoantibodies, but does not directly target the underlying pathophysiology. Rituximab and steroids allow for immunosuppression and decreased production of antibodies but do not inhibit interaction between existing autoantibodies and ADAMTS13. Caplacizumab, a novel agent, has been shown to provide an important therapeutic role in management of iTTP by targeting a portion of the underlying pathophysiology at play. Caplacizumab is a humanized bivalent variable-domain-only immunoglobulin fragment that targets the A1 domain of VWF (30). It prevents VWF from functioning in platelet recruitment *via* binding of its A1 domain to the GPIb-IX-V platelet receptors, directly targeting the underlying pathophysiology of microthrombosis in TTP. Results of 2 randomized controlled trials have led to approval of caplacizumab for the treatment of adults with iTTP in Europe in 2018 and FDA approval in 2019 (31, 32). International Society on Thrombosis and Haemostasis (ISTH) guidelines support the use of upfront caplacizumab for management of iTTP in adults based on the clinical trials described, despite some potential bleeding risks, still to be defined (33, 34). As expected, our case 2 patient with confirmed iTTP, received TPE and concomitant immunosuppressive therapy. She received 7 days of TPE, with high-dose glucocorticoids (subsequently weaned over ~4 months) and a course of rituximab (375 mg/m^2^/dose x 4 weekly doses), an anti-CD20 monoclonal antibody. She demonstrated a rapid response to therapies, with resolution of anemia and thrombocytopenia within 1 week and normalization of ADAMTS13 activity within 2 weeks and resolving hemolysis over the following weeks. She has maintained a durable response to therapy with no evidence of recurrence >6 months from diagnosis.

However, our patient in case 1 was diagnosed with iTTP prior to the results of molecular testing. As a result, immediate TPE and immunosuppression including glucocorticoid therapy, rituximab, and cyclosporine were initiated. He achieved a detectable ADAMTS13 level while on TPE which was short-lived. He did not appear to respond to immunosuppressive therapy and also suffered significant unwanted side effects secondary to high doses of glucocorticoids. Furthermore, irreversible immunosuppressive therapies placed him at prolonged infectious risk prior to his diagnosis of cTTP, which may have been prevented with earlier genetic testing. Ultimately, caplacizumab was initiated. He never achieved detectable ADAMTS13 activity level while receiving caplacizumab therapy; however, interestingly he exhibited significant improvement in hemolytic anemia, thrombocytopenia, and overall functional status. This improvement was short lived, and with cessation of caplacizumab therapy, he again developed clinical signs and symptoms of recurrent TTP.

Alternatively, the mainstay of therapy for cTTP is replacement of the absent or deficient ADAMTS13 enzyme, which has traditionally been done utilizing FFP infusions. However, this can be cumbersome for patients to maintain long-term, requiring frequent administrations within a clinic or hospital setting, and placing patients at risk for antibody development and allergic reactions due to repeated exposures. In addition, an international registry-based review has shown that despite regular prophylaxis with FFP patients remain at relatively high risk for recurrent episodes of TTP, particularly pediatric patients (35). Several studies have explored the use of intermediate-purity factor VIII concentrates for both treatment and prophylaxis in congenital TTP, as certain commercially available products have been shown to contain high amounts of ADAMTS13. Use of such products would potentially allow for less frequent infusions, in-home administration, smaller infusion volumes, and decreased risk of blood-born infections or transfusion-related reactions when compared to standard FFP administration. Several case series report their successful use for this population (36, 37). Studies have analyzed functional and immunoreactive ADAMTS13 in several commercially available products in the US and EU and identified two products (Koate-DVI^®^ and Alphanate^®^) with high ADAMTS13 activity: 900% and 200%, respectively, compared to activity of 100% in normal pooled plasma, making these products a viable alternative for patients with cTTP (38). Once molecular diagnosis of cTTP was made for our patient in case 1, immunosuppressive therapy was discontinued and a replacement approach was utilized – initially with fresh frozen plasma (FFP), and subsequently with the ADAMTS13-containing factor 8 product Koate-DVI^®^. He remained clinically asymptomatic without laboratory evidence of recurrent cytopenias or MAHA and was ultimately transitioned to a home prophylactic regimen of Koate-DVI^®^ 50 Units/kg twice weekly, with trough ADAMTS13 activity ranging from 8% to 29%.

More recently, a phase 1 study exploring the safety of the use of BAX930 a recombinant ADAMTS-13 (rADAMTS-13) in patients with severe cTTP demonstrated tolerance and safety, as well as pharmacokinetic profile comparable to that of plasma infusions (39). These results have led to an ongoing phase 3 open label multi-center trial evaluating the safety and efficacy of rADAMTS-13 for prophylaxis as well as treatment for pediatric and adult patients with confirmed severe cTTP (*NCT03393975*).

### Conclusion

Because childhood onset TTP is incredibly rare, there are frequent delays in diagnosis of both iTTP and cTTP, which can lead to inappropriate management or delay in appropriate management, potentially resulting in severe consequences. TTP, particularly when presenting in childhood, may be mistaken for other autoimmune phenomena such as ITP, Evans syndrome, or HUS. As the management of these disorders differs widely, prompt and accurate diagnosis is crucial – first with ADAMTS13 activity measurement and second with ADAMTS13 inhibitor assessment. As demonstrated in Case 1, molecular testing may ultimately be the key to distinguishing cTTP from iTTP, in the setting of uninterpretable inhibitor testing and unclear clinical phenotype, in order to more expeditiously provide the appropriate management for these children and adolescents with TTP.

## Data Availability Statement

The original contributions presented in the study are included in the article/supplementary material. Further inquiries can be directed to the corresponding author.

## Ethics Statement

Ethical review and approval was not required for the study on human participants in accordance with the local legislation and institutional requirements. Written informed consent to participate in this study was provided by the participants’ legal guardian/next of kin.

## Author Contributions

CK, SK, JE, and AG collected and verified clinical data. CK drafted the manuscript. CK, SK, HH, JE, JD, and AG have all reviewed and edited the manuscript. All authors contributed to the article and approved the submitted version.

## Conflict of Interest

SK receives honoraria from BioMarin. JD receives consultancy fees, honoraria and research support from Novartis, honoraria from Dova and Amgen and royalties from Uptodate.

The remaining authors declare that the research was conducted in the absence of any commercial or financial relationships that could be construed as a potential conflict of interest.

## Publisher’s Note

All claims expressed in this article are solely those of the authors and do not necessarily represent those of their affiliated organizations, or those of the publisher, the editors and the reviewers. Any product that may be evaluated in this article, or claim that may be made by its manufacturer, is not guaranteed or endorsed by the publisher.
